# Supplementary material for: Validation testing to determine the sensitivity of lateral flow testing for asymptomatic SARS-CoV-2 detection in low prevalence settings: Testing frequency and public health messaging is key
Source: PLoS Biol. 2021 Apr 29;19(4):e3001216. doi: 10.1371/journal.pbio.3001216 (PMC8112643; doi:10.1371/journal.pbio.3001216)
Supplement: Attachment — Submitted filename: Reviewers_comments.docx [file pbio.3001216.s002.docx]

We would like to thank the editor and reviewers for their critique and suggestions, which have allowed us to greatly improve our manuscript. We have incorporated these changes, which are detailed on a point-by-point basis below.

**In particular, reviewer #1's major concern is the lack of empirical validation of the presence of infectious virus in the tested samples. We understand that this may not be possible to do retrospectively, but you should then discuss the implications and limitations of this study. For this issue, the Academic Editor also recommends to find in the literature examples of measurements of the same samples of SARS-CoV-2 for infectivity and for the levels of RNA as measured by PCR.**

Whilst we are unable to validate viral titre due to the barriers to culturing SARS-CoV-2, we have included references to studies (e.g. Public Health England’s validation study) who have done so. We also feel that the validation using the Qnostic panel addresses this, albeit in a less-than-ideal fashion.

**Both reviewers also agree that you should add a discussion, add references, and provide clarifications throughout the manuscript. Reviewer #1 also says that it is critical to determine the LOD of both devices in parallel with the same panel to make sure the PCR threshold is accurately estimated, as this is crucial for the theoretical evaluation and the overall conclusions of the study. Please address the reviewers' concerns.**

We have included a fully referenced discussion, and included additional details regarding the limit of detection of the Innova LFD platform. The Qnostics panel is composed of irradiated virus and so is not detectable on the LFD platform. This is the very reason we chose the approach we did, and also now point out in the discussion that our extrapolation data is in line with other published comparative LoD data.

**In order to make the manuscript more accessible to a general readership, we think that it would help adding a few sentences at the end of the abstract to explain the implications that Lateral Flow Devices do not detect infections presenting with PCR Ct values over 29-30, what % of this population was detected and undetected, and if any recommendation can be gleaned from it. We also recommend a more declarative title modifying it to include an idea of the conclusions of the work.**

We have included a summary sentence intended for a lay audience at the end of the abstract, and have revised the title to include our main recommendations.

Major
**1. My major concern regarding this study is the lack of empirical validation of the presence of infectious virus in the tested samples. The authors conclude that the LFD could not detect infections with Ct values over Ct 29-30. Based on previous studies we know that this is in line with the typical threshold of samples containing infectious virus, but it would have been much stronger if the authors could provide this evidence. Ct values can differ drastically between labs and different PCR platforms, and therefore it would be preferable to empirically determine the correlation between infectivity and Ct values for this particular study. I understand that these experiments are very challenging and that they might not be possible retrospectively, but at the minimum the authors should discuss the true implications and limitations of this study, which is currently lacking in the manuscript. Particularly, a discussion on the application of LFD for SARS-CoV-2 screening is needed as it can still be used as an effective tool despite the lower sensitivity in comparison to PCR. Please cite previous studies accordingly throughout the manuscript.**

Please see our point above. Whilst we are unable to validate viral titre due to the barriers to culturing SARS-CoV-2, we have included references to studies (e.g. Public Health England’s validation study) who have done so. We also feel that the validation using the Qnostic panel addresses this, albeit in a less-than-ideal fashion.

**2. The LOD experiments require some clarifications. The authors tested the Qnostics Q-panel to estimate the Ct value that would match the LOD of the LFD (100 copies/mL). However, it seems striking to me that not all 3 PCR targets were able to consistently detect the panel at 100 copies/mL, while the LOD for the PCR should be much more sensitive than the LFD. It looks like the panel consists of inactivated virus, but I wonder if the reported concentrations are for PFU/mL or RNA copies/mL. This would make a huge difference as the authors report in the introduction that the LOD of the LFD is 100 PFU/mL or 100.000 RNA copies/mL. It would be critical to determine the LOD of both devices in parallel with the same panel to make sure the PCR threshold is accurately estimated, as this is crucial for the theoretical evaluation and the overall conclusions of the study. Please also clarify whether the LOD was tested in 3 (main text) or 5 (legend table 3) replicates.**

Please see our point above. The Qnostics panel is composed of irradiated virus and so is not detectable on the LFD platform. This is the very reason we chose the approach we did, and also now point out in the discussion that our extrapolation data is in line with other published comparative LoD data.

**3. I would suggest to extend the introduction by (1) describing background on the use and performance of lateral flow tests for SARS-CoV-2 screening of asymptomatic individuals (key publications are missing), (2) stating the overall aims of the study more clearly at the end, (3) summarizing the main findings.**

We have now included reference to further asymptomatic screening publications throughout the manuscript which were not actually published when we first performed and drafted this report. We have added the overall aims and main findings/conclusions to the introduction.

**4. The methods currently lack (1) an ethics statement, and (2) brief description to the Innova, RNA extraction, and TaqPath protocols.**

An ethics statement is now included. We have included brief descriptions, and referred to the manufacturer’s protocols where appropriate.

Minor
**1. Title suggestion: Validation of the effectiveness of the Innova lateral flow device for asymptomatic SARS-CoV-2 detection in low prevalence settings. Perhaps even making the title more specific by replacing "effectiveness" by "sensitivity" or another more specific term.**

We altered the title, including replacement of effectiveness with sensitivity.

**2. Line 91: Please specify how much the sample was diluted by adding 500 uL of water. Was this dilution factor taken into account when comparing LFD and PCR results?**

We have added further details of the amount of water added to samples. As this does not affect the amount total RNA present in the solution, and occurs after the sample has been loaded onto the LFD, it does not affect comparability.

**3. Line 173: please rephrase**

We have rephrased line 173.

**4. Fig1: remove zeros after decimal on Y-axis. X-axis label should read "number of tested samples"**

We have corrected Figure 1.

**5. Line 35 and Line 190: Ct values can differ significantly between different labs and PCR platforms. Please specify that the observed Ct value threshold is specific for the used PCR platform.**

We have included details of our results being specific to our lab and assay.


**1. The first sentence needs a reference.**

We have referenced the first sentence.

**2. Can the authors provide some more context about why the Innova LFD was 'principal' in being rolled out? This isn't immediately obvious for a non-UK audience.**

This isn’t immediately obvious even within the UK, as procurement decisions are driven by ministerial approval. Without relying on conjecture, we have posited that the Innova platform was simply the first to pass a 4 phase validation, though this may obviously be driven by things like availability, cost, and logistical factors that we are not able to cite.

**3. 'Data from the city council shows that 71,684 LFD tests were performed alongside 51,855 gold-standard PCR tests' - this makes it sounds like tests were performed at the same time in the same individuals - can the authors clarify the number of individuals tested. Not clear from this sentence.**

We have clarified the number of individuals tested in parentheses.

**4. Lines 49-50 - needs ref.**

We have added a reference. **5. Line 55 - what is the FALCON trial?**

We have added a reference providing details of the NIHR FALCON clinical trial. **6. Lines 56-57 needs references.**

We have added references.
 **7. Lines 60-61 - why can't the authors access this information - bit speculative otherwise.**

We have now reworded this to state the Ct values were not generated with the Thermo TaqPath assay. Apologies for the initial clumsy wording


**8. Lines 79-80 - what was the result recording app? More detail required here.**

The results app was provided to us by DHSC, we have reworded this section to clarify this.

**9. Please provide information on what 'pillar' laboratories are - again, not clear to a non-UK audience.**

We have provided the definition of Pillar 2 testing laboratory.

**10. Line 86: Why were 90 samples selected - was this figure derived statistically?**

90 samples were used as the automated extraction and processing pipleline is based around a 96 well plate format, including space for positive and negative controls. We have added details of this into the manuscript.

**11. Line 86: How do the authors know that the solution is saline? Please reference the Innova IFU (I'm aware that other LFD solutions are not saline).**

We have removed reference to the buffer being saline, and simply call if ‘sample buffer’

**12. Line 99 - should read 720/7187**

We have corrected this.


**13. Line 119: please provide criteria for deeming a sample void.**

We have included a line stating that void samples are defined according to the manufacturer’s specifications. At the start of the testing, some cartridges would not correctly wick the sample through to the test area. We empirically observed that this is ocassionally due to the omission of a silica gel pack within the cartridge packaging, potentially increasing the relative humidity within the package, and preventing efficient diffusion. We adjusted our testing protocol, and used additional cartridges where possible, which eliminated any void samples very early on. As this happened for such a small number, and as only a very limited number of cartridges performed in this manner, we do not think it is appropriate to include full details of this in the manuscript.

**14. Line 144: RT-PR should be RT-PCR**

We have corrected this mistake.

**15. Line 159: 'From our data the Lateral Flow Device test yield is 2.8 per 10,000 (0.3 to 10 per 10,000).' - not clear what the units here are? Tests?**

We have added a unit to make this clearer.

**16. Line 190: 'However it is incapable of detecting infection at comparable PCR Ct values of 30 and over.' - this needs qualifying to say that this is only based on an analysis using the TaqPath assay - it may be different with other assays.**

We have specified that our results are based on the TaqPath Assay.

**I was a bit surprised not to see a Discussion here - there are obviously lots of facets to discuss (implications of false pos/neg; logistical roll out of tests; impact of variants of concern on LFD detection; frequency of testing). The authors should include a broader discussion of their findings here - not enough just to have a brief concussion section.**

We have added a discussion.
